# Medical Society of the County of Erie

**Published:** 1903-08

**Authors:** Franklin C. Gram


### SOCIETY PROCEEDINGS.

### Medical Society of the County of Erie.

*Semiannual Meeting, June 9, 1903.*

REPORTED BY FRANKLIN C. GRAM, M. D., Secretary.

The eighty-second semiannual meeting of the Medical Society of the County of Erie, was held June 9, 1903, at 10 o'clock a.m.. in the Y.M.C.A. parlors, Buffalo; the president, Dr. ERNEST WENDE in the chair.

The minutes of the previous meeting were read and adopted.

Dr. William Warren Potter, chairman of the membership committee, presented the names of Roy G. Strong and William Hobson Heath. They were elected to membership.

The special committee appointed to investigate the matter referred to in the annual report of the board of censors, regarding Dr. C. H. Woodard, reported through its chairman, Dr. John H.

Grant. The secretary of the society read Dr. Woodard's resignation as a member of the society. On motion of Dr. Henry R. Hopkins, the report of the committee and Dr. Woodard's resignation were referred to the board of censors.

Dr. Irving W. Potter, chairman of the board of censors, presented the following report:

*To the Medical Society of the County of Erie:*

The board of censors during the past six months have investigated, upon complaint, the following cases:

Prof. DeNoird doing business in the Ellicott Square building was warned of being in violation of the law in advertising himself as "Doctor," and upon his promising to cease and leave the city, he was not prosecuted. He subsequently left town in accordance with his promise.

Frank Smiseszny, of 81 Mills Street, also promised to stop practising medicine after being told he would be prosecuted if he so continued.

Through the efforts of the *Morning Review,* Antonius J. Weichers was arrested, tried, and convicted in the Supreme Court of conspiracy to defraud, and sentenced to nine months' imprisonment in Erie County Penitentiary. At the request of the district attorney, the censors had the contents of two bottles of medicine given by Antonius to his patients examined by the city chemist, and his report was given to the district attorney for use in the trial.

On March 30, 1903, a Mrs. Tyler made arrangements to open at Concert Hall, and pursue a similar line of work to that done by Antonius. This board engaged I. L. Sherman to gather evidence of what she did, and he has made his report to the board. It appears from this that Mrs. Tyler was backed by Dr. S. H. Warren, who stated that he was the physician in charge of the Tyler Magnetic Sanitarium at 78 Niagara Street. Upon watching Mrs. Tyler for a week Mr. Sherman informed her she would have to stop or she would be arrested and prosecuted. She closed up and no more complaints have been made concerning her.

A complaint was made against S. T. Lee for practising medicine without authority, and upon investigation, the evidence seemed strong enough to convict him. He was arrested and tried in Police Court, but was discharged. This was Mr. Lee's second offense, he previously having been fined $100.00.

A "Dr." Bridgewater, who followed Antonius in the house where the latter conducted his fraudulent work, and undertook to continue it on similar lines, was arrested, tried, convicted and fined $50.00 for the illegal practice of medicine. He paid the fine, which, we understand, has been turned over to the treasurer of this society.

This board desires to express its gratitude to the district attorney and his assistants for the manner in which they have at all times aided this society.

We also wish to acknowledge our indebtedness to our counsel, Mr. John Lord O'Brian, for his services, and would respectfully recommend that the board be given authority to draw upon the treasurer for such compensation as the board may approve for payment of counsel, and to meet I. L. Sherman's bill of $25.00 for service in the Tyler case.

Respectfully submitted,

Irving W. Potter, *Chairman.*
Francis E. Fronczak,
H. R. Hopkins.

Buffalo, June 9, 1903.

On motion of Dr. Hopkins the report was adopted and the recommendations therein approved.

Dr. John H. Grant moved that this society request the board of regents of the State of New York to revoke the license of one Dr. Franklin Stuart Temple, who had been associated with Antonius, now serving a sentence in the Erie County Penitentiary. The motion was adopted.

The society then listened to interesting and instructive addresses, as follows: Soul health, by Rev. O. P. Gifford, D.D.; The doctor from the lawyer's standpoint, by John W. Fisher, Esq., of the Buffalo Bar; The physician as an advertising medium, by Willis G. Gregory, M.D., Ph. G.; The physiology of the internal secretions, by F. C. Busch, M.D.

A vote of thanks was tendered the speakers, after which the society adjourned.

